# Imaging technologies for preclinical models of bone and joint disorders

**DOI:** 10.1186/2191-219X-1-11

**Published:** 2011-07-29

**Authors:** Jordi L Tremoleda, Magdy Khalil, Luke L Gompels, Marzena Wylezinska-Arridge, Tonia Vincent, Willy Gsell

**Affiliations:** 1 Biological Imaging Centre (BIC), Medical Research Council (MRC) Clinical Sciences Centre, Imperial College London, Hammersmith Campus, London W12 0NN, UK; 2 Faculty of Medicine, Kennedy Institute of Rheumatology, Imperial College London, London W6 8LH, UK

**Keywords:** imaging, animal models, bone, cartilage, micro-CT

## Abstract

Preclinical models for musculoskeletal disorders are critical for understanding the pathogenesis of bone and joint disorders in humans and the development of effective therapies. The assessment of these models primarily relies on morphological analysis which remains time consuming and costly, requiring large numbers of animals to be tested through different stages of the disease. The implementation of preclinical imaging represents a keystone in the refinement of animal models allowing longitudinal studies and enabling a powerful, non-invasive and clinically translatable way for monitoring disease progression in real time. Our aim is to highlight examples that demonstrate the advantages and limitations of different imaging modalities including magnetic resonance imaging (MRI), computed tomography (CT), positron emission tomography (PET), single-photon emission computed tomography (SPECT) and optical imaging. All of which are in current use in preclinical skeletal research. MRI can provide high resolution of soft tissue structures, but imaging requires comparatively long acquisition times; hence, animals require long-term anaesthesia. CT is extensively used in bone and joint disorders providing excellent spatial resolution and good contrast for bone imaging. Despite its excellent structural assessment of mineralized structures, CT does not provide *in vivo *functional information of ongoing biological processes. Nuclear medicine is a very promising tool for investigating functional and molecular processes *in vivo *with new tracers becoming available as biomarkers. The combined use of imaging modalities also holds significant potential for the assessment of disease pathogenesis in animal models of musculoskeletal disorders, minimising the use of conventional invasive methods and animal redundancy.

## Introduction

Bone and joint disorders impose an enormous social and economic burden on society, causing disability and substantial patient morbidity. There is a rising demand for developing effective therapies to improve such conditions [1]. The process of discovering and bringing drugs to the clinics remains long and expensive; thus, improving its efficiency remains a major target. Preclinical testing plays a major role in this process enabling powerful and clinically translatable methods for monitoring disease progression and testing drug candidates. Imaging is becoming an important key technology in this process, with its ability to deliver non-invasive and quantitative cellular and molecular information that can access the mechanisms of drug action. Thus, imaging is one of the most promising technologies for improving the drug development process, facilitating translation between preclinical and clinical findings [2]. In the last few years, many large pharmaceutical companies and research institutions are implementing imaging at critical stages of their preclinical studies.

With advances in transgenics and animal models for human disease, researchers are increasingly using imaging not only for drug discovery but also for phenotyping and understanding the pathophysiology of disease. Its use has represented a keystone in the refinement of animal models, allowing longitudinal studies and enabling a powerful, non-invasive and clinically translatable way for monitoring disease progression in real time. For many years, studies in animal models relied on histological analysis of tissues and/or organs post-mortem. These destructive methods limited the ability of researchers to study the progression of the disease on a single animal serially over time as well as assessing therapeutic efficiency overtime.

Imaging technologies provide good tools for assessing anatomical, morphological, physiological and functional parameters and molecular and cellular processes in animal models of disease. Imaging protocols and agents have been developed to enable good spatial resolution for the physiological and functional properties of targeted tissue, including blood flow, tissue permeability, metabolism, tissue density, cellular proliferation and oxygenation. Currently, imaging is increasingly being implemented at the late stage of preclinical development through to the clinical phases, taking a major role in the validation of specific candidate drug or treatment regime. These non-invasive technologies allow for the combined use of different modalities to obtain multiple physiological and functional parameters from a single animal study. This optimizes greatly the power of such animal studies and the efficacy of experimental readouts.

The objective of this review is to familiarise the reader with a selection from the range of imaging possibilities that are available for assessing bone and joint disorders in animal models. We will review the technologies available, discuss their current applications and address their challenges and future implications for refinement of musculoskeletal animal models.

## Imaging technologies

### Micro-computed tomographic imaging

#### Overview of CT technology

The development of dedicated imaging equipment for small animals and, in particular, the implementation of computed tomography (CT) have revolutionised the use of animal models in musculoskeletal research, becoming the gold standard for evaluation of bone morphology and micro-architecture in animal models [3,4]. While histomorphometric assessment has been extensively used as the main standard for investigating bone architecture, the development of 3D imaging techniques such as CT have provided an accurate non-invasive tool for directly measuring bone architecture. Indeed, since the development of clinical CT, the examination of small animals for research using purpose built CT has rapidly advanced providing high-quality resolution and fast reconstruction and assessment protocols for preclinical applications [5-7].

**Figure 1:**
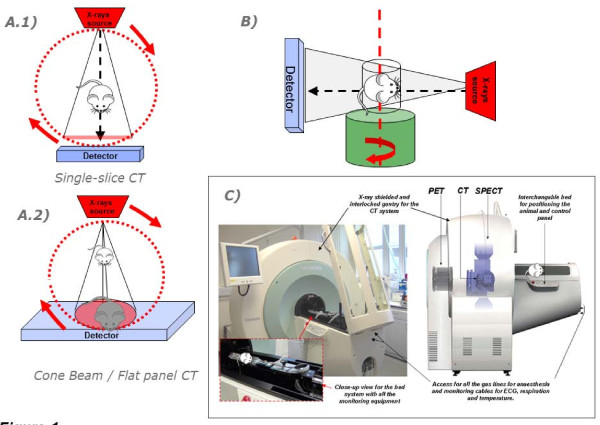
**Diagrams showing the different micro-CT designs for imaging animal models**. In the systems shown in image (**A**, 1 and 2), the animal is placed in the centre of the set-up and the gantry carrying the detector and the X-rays source is rotated around it. This is the common setup used in *in vivo *preclinical imaging. A fan-shaped beam system fitted with a single-slice detector and cone beam system fitted with flat-panel detector are displayed in (A, 1 and 2), respectively. In the system (**B**), the specimen is placed on a stand that rotates within its own axis in the course of the beam. Image (**C**) shows a multimodality scanner in which the CT is integrated with PET and SPECT systems (Inveon System Siemens Medical Solutions, Knoxville, TN, USA). This versatile system allows unified PET, SPECT and CT data acquisition. The CT system has an automated zoom control which allows the operator to adjust the field of view and magnification.

Micro-CT uses X-ray attenuation data acquired at multiple viewing angles to reconstruct a 3D representation of the imaged specimen, characterising the spatial distribution of the material density [8,9]. Currently available micro-CT scanners can achieve high resolution with an isotopic voxel size of as low as a few micrometres (down to 5 μm; although new generation on nano-scanners can go down below 1 μm) [10]. There are, broadly speaking, two different micro-CT construction systems, one type in which the examined object is placed in the centre and the X-ray detector and radiation source is mounted in a gantry that rotates around it; in this system, the geometrical magnification is defined by the source-detector distance. This set-up is the one most commonly adapted for animal scanners. In the second type of CT scanner, the object is rotated within the course of the X-ray beam and the set-up allows the free positioning of the sample between the detector and the source, allowing the adjustment of the magnification level. This second construction is more often utilised in *ex vivo *custom-built systems. There are also differences on the beam geometry of the X-ray source used. Images can be acquired by using either a fan-shaped beam in which data are acquired through dynamic acquisition plane by plane or by a cone beam - this is also called 'volume-CT' [11] where the scanned subject is captured completely (based on the axial extent of the CT field of view) in one rotation, speeding up the imaging process. Furthermore, micro-CT systems can be fitted with a flat-panel-based detector system with slip ring technology that allows for very high-speed data collection [12]. The rapid acquisition times come at the expense of compromising spatial resolution, but this may be justifiable for *in vivo *applications that require rapid scan times such as perfusion imaging and high-throughput imaging. Similarly, tissue contrast enhancement can be induced by using a dual-energy X-ray computed tomography method in which the projection data are acquired by using two different X-ray spectra [13]. Figure 1 illustrates the different micro-CT systems technologies.

Current availability of multi-modality imaging platforms which can provide integrated positron emission tomography (PET)/single-photon emission computed tomography (SPECT)/computed tomography (CT) imaging and analysis are extremely useful for co-registering images within the same gantry, facilitating a multi-modal imaging approach within the same animal *in vivo *at the same time points during disease evolution [14]. Moreover, rapid image acquisition can also be facilitated by gating signal acquisition to cardiac and respiratory cycles by utilising a high-speed shutter system that allows image times as short as 10 ms [15].

#### Micro-CT imaging applications of preclinical models for bone and joint disorders

**Figure 2:**
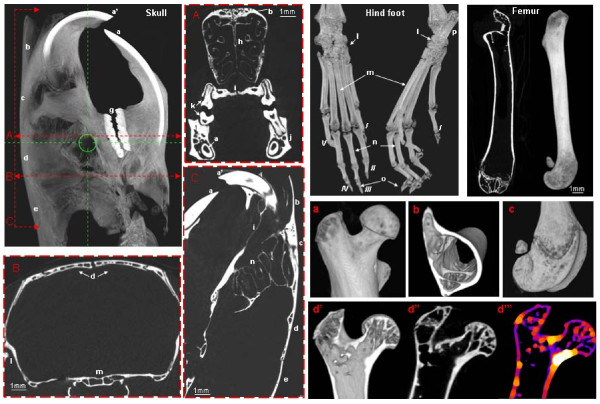
**Micro-CT images acquired *ex vivo *from Nude BALB/c mouse skull (3D-volume rendering image)**. Displaying different anatomical regions: (a, a') lower and upper incisor tooth, (b) nasal bone, (c) frontal bone, (d) parietal bone, (e) intraparietal bone, (f) right mandible and (g) molar tooth. (A, B) Coronal views of cranial and caudal 34 areas of the skull, respectively, displaying in image A: (a) roots of the lower incisor, (b) frontal bone, (h) nasal septum, (i) maxillar/palatine bone, (j) mandible and (k) roots of molar tooth; in B: (d) parietal bones, (l) temporal line and (m) basis sphenoid bone. (C) Sagittal view of the skull displaying incisor roots (a, a'), nasal, frontal, parietal, intraparietal bones (b, c, d, e) and endoturbine structures (n). Micro-CT images from Nude BALB/c mouse hind foot (3D-volume rendering) showing the *I-V *phalanges, (l) tarso-crural joint, (m) metatarsal bone, (n) digital bone, (o) claw, (p) calcaneous bone. Examples of micro-CT C57BL/6 mouse femur showing coronal view and 3D-volume rendering image. (a, b, c) Close views of the proximal epiphysis (head and greater trochanter), metaphysis area displaying cortical and trabecular bone and the femur condyles, respectively. (d) Images displaying a sagittal 3D and 2D cross section of the proximal epiphysis (d', d'') and a sagittal cross view showing a map of the bone thickness with higher density areas displayed as white (femoral neck). All the samples were acquired at 80 kVp, 500 μA and with a pixel size of 9.5 μm; images were reconstructed in Hounsfield units (HU) and processed with ImageJ (NIH, Bethesda, MD, USA).

Micro-CT is extensively used to investigate the structure and density of bone in rodents. Micro-CT has high spatial resolution and contrast for imaging mineralised tissues and the ability to qualitatively and quantitatively assess 3D bone structures. This allows measurements of trabecular morphology such as thickness and separation. Micro-CT has been used for a wide range of bone studies, including bone anatomy and density to assess bone repair during fracture healing [16]; bone resorption, remodelling and regeneration [17]; bone neoplasm and metastases [18] and bone changes influenced by metabolic disorders, e.g. osteoporosis [19] and the characterisation of skeletal phenotypes from different mouse strains [20]. Figure 2 provides an example of different imaging acquisitions of various mouse bones used for skeleton phenotypes and bone repair studies.

The high isotropic resolution can also provide good information on trabecular bone spatial orientation patterns, density, and geometry and growth plate morphology. While much finer detailed imaging can be achieved in *ex vivo *samples, micro-CT imaging has proven very valuable for *in vivo *longitudinal studies. At least a 100 μm isotropic spatial resolution can be effectively achieved within a safe and reasonable acquisition time through longitudinal *in vivo *studies [21]. Some studies have reported *in vivo *isotropic resolutions down to 15 μm in serial imaging sessions using rats (seven sessions of 10 min acquisitions with an absorbed dose of 0.5 Gy-CTDI) [22], but there are concerns about the amount of ionizing radiation delivered during repetitive *in vivo *scanning. This radiation may introduce unwanted effects on the tissues or processes of interest or have an adverse effect on animal welfare [23].

**Figure 3:**
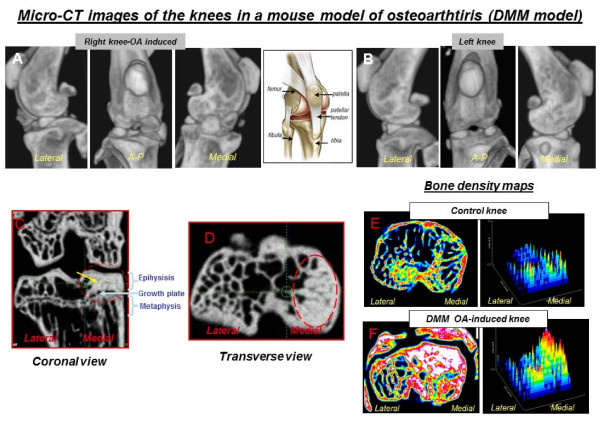
**Computed tomography imaging of the knees in a mouse model of osteoarthritis (DMM model)**. Images of non-injured mouse knee (left knee, **B**) and 2 months after surgical induction of osteoarthritis (DMM model; right knee, **A**) were acquired *ex vivo *at 80 kVp, 500 μA and with a pixel size of 35 μm. Series (A) and (B) show 3D surface rendering CT lateral, anterior/posterior and medial views of the DMM-knee displaying OA-related derangements of the knee morphology 35 (remodelling of subchondral bone, hypertrophic calcifications; A), and of a non-injured knee (B), respectively. Images were analysed for differences in subchondral sclerosis in the epiphysis: (**C**) coronal and (**D**transverse views. Bone density maps show different densities throughout the subchondral weight-bearing lateral and medial regions of the tibial plateau in a controlled non-injured knee (**E**) versus a DMM OA-induced knee (**F**). Notice the high-density area in the medial region (DMM-injury site). Bone density was measured from micro-CT images (voxel size of 35 μm) normalise to HU units, and images were processed with ImageJ (NIH).

Micro-CT imaging has also proven to be very valuable in investigating morphometric changes in joints in osteoarthritis (OA) animal models [24-26]; it has been successfully applied to study changes in the subchondral bone architecture in excised osteoarthritic knees in collagenase-induced OA mice [27] and in surgical destabilisation models [28]. Figure 3 shows micro-CT athrograms of mouse knees from the surgical destabilisation of the medial meniscus (DMM) model. Recently, Stok and collaborators [29], by imaging excised knees *ex vivo*, showed how changes in subchondral bone seem to be inversely correlated to the ongoing degenerative changes in the articular cartilage in STR/ort mice (naturally occurring OA). Indeed, micro-CT imaging provides quantitative and qualitative 2D and 3D assessments of bony structures in the osteoarthritic joint such as subchondral bone morphology and bone mineral density, trabecular bone patterns, meniscus morphology, heterotopic ossification and subchondral cyst formation [30]. Figure 3 shows micro-CT athrograms of mouse knees in C57BL/6 mice, 8 weeks after surgical destabilisation of the medial meniscus to investigate changes in the subchondral bone (Tremoleda et al., unpublished data). Interestingly, such technology has also been successfully incorporated to monitor the progression of OA in longitudinal studies in rats [31].

One of the major challenges for imaging osteoarthitis models is that the micro-CT has low sensitivity for soft tissue; hence, compromising the visualisation of the degenerative changes in the articular cartilage. Contrast agents can be used to enhance the contrast resolution of CT imaging, and there have been a few attempts to indirectly visualise the cartilage morphology using these agents in *ex vivo *samples [32,33]. To compensate for the poor radio-opacity of the cartilage, tissues to be imaged are equilibrated with an ionic contrast agent which is taken up by the cartilage matrix and distributed inversely to the density of the negatively charged glycosaminoglycans (GAGs). This contrast-enhanced CT technique which is based on the detection of the equilibrium partitioning of an ionic contrast agent (EPIC-micro-CT) provides good 3D characterisation of the articular surface morphology and ligament insertions *in situ *in *ex vivo *rabbit knees [31]. This technique allows for the monitoring of surface contours and healing processes in injured articular cartilage in defect repair studies as well as changes in cartilage thickness. Changes to other anatomical features such as ligaments and non-calcified menisci can also be detected. While the use of this methodology has great potential for assessing cartilage degeneration in *in vivo *longitudinal studies, the thin layer of articular cartilage and the small joint space in mice combined with diffusion variability between contrast agents remain challenges for accurate quantification of signal change.

### Magnetic resonance imaging

#### Overview of the MRI technology

Magnetic resonance imaging (MRI) is a non-ionizing 3D imaging technique that has advantages over other methods that depend on ionizing radiation such as CT, SPECT and PET. It remains one of the main imaging applications of choice for assessment of musculoskeletal tissue structures such as tendons, cartilage, menisci and ligaments in the clinical setting and has also being successfully tested in animal models (mice [34], rats [35] and rabbits [36]).

MRI technology uses the magnetic properties of atoms and molecules of tissues to be imaged and their interactions with both a large external magnetic field and radiowaves. The proton ^1^H is the nucleus mostly used for anatomical imaging because of its abundance of soft tissue structures where there is a high water content. MRI technology utilises a powerful magnetic field (non-ionizing radiation) to align the nuclear magnetization of hydrogen atoms, and then, radio frequency (RF) pulses (through RF coils) are used to systematically alter this alignment, causing the hydrogen nuclei to produce a rotating magnetic field that is detectable by the scanner. The application of radio frequency pulses rotates the magnetization by 90°. Then, the magnetization returns to its initial value, and the rate at which the magnetization decays away is characterised by two relaxation times named as T1 and T2. These signals are then digitalised and processed to build up enough information to construct an image of the targeted body area. It is this relationship between field strength and frequency implemented onto the hydrogen atoms within the tissue that allows the use of nuclear magnetic resonance for imaging.

#### MRI applications for imaging preclinical models for bone and joint disorders

MRI assessment of bone structure has remained a challenge due to its composite biomaterial characteristics. Bone is made up of an organic substrate (mostly collagen type I approximately 40% by volume) and mineral crystals of hydroxyapatite (approximately 45%). The remaining volume is occupied by water (approximately 15%). Due to this limited water composition, proton imaging of bone remains challenging. Advanced micro-MRI systems have been used successfully to image trabecular bone in rats *in vivo *[37]. These methods rely on indirectly imaging the trabecular bone structure that appears as a signal void surrounded by high-intensity signal from the fatty bone marrow. Because of the small trabecular thickness in small animals (50 to 100 μm in rats; 40 to 60 μm in mice), the resolution requirements are more stringent with a high risk of overestimation of the trabecular thickness depending on the volume of the trabeculae and therefore an adverse signal-to-noise ratio - reinforcing the need for longer acquisition times.

In the preclinical setting, the acquisition of high-resolution MRI within an acceptable time frame remains an important drawback due to the relatively small size of anatomical structures in rodents; long acquisition is needed to generate sufficient isotropic (3D) resolution with a small-bore MRI and with high field strength. This needs to be performed with the animal under general anaesthesia.

To overcome the limited detection sensitivity and to increase the signal-to-noise ratio, enhanced pulse sequences have been developed. These allow adequate sample volumes to be scanned with scan times maintained within the limits of *in vivo *tolerance. 3D gradient-echo sequences allow for a shorter TE than spin-echo or fast spin-echo sequences, allowing for faster data acquisition compared to fast spin-echo sequences, and therefore allow more data averaging within a fixed duration of scan time [38].

**Figure 4:**
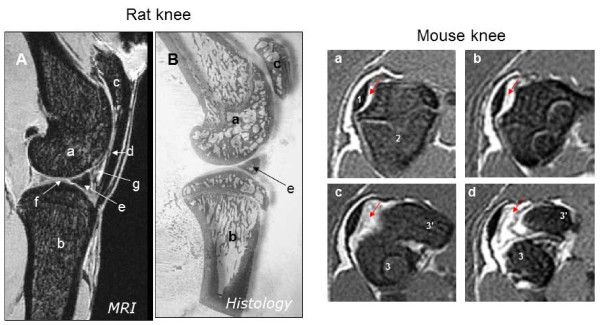
**MRI images of a rat (Wistar) and mouse (C57BL/6) knee joint**. (A) 3D spin echo MR image (117 × 114 × 144 μm) of a rat knee *ex vivo *displaying the anatomical landmarks of the articular joint: a = femur condyle, b = tibia, c = patella, d = patellar ligament, e = meniscus, f = articular cartilage and g = intrapatellar fat pad. (B) Histological image of the knee joint. The MR images provided an excellent visualisation of the rat knee anatomy, with detailed observations on the subchondral bone and in the articular synovial space. (a, b, c, d) Sequential fast spin echo multi-slices images (axial views from proximal to palmar) from the proximal region of the mouse knee (512 × 512 μm). The MR images displayed the bones of the area of knee joint (1 = patella; 2 = femur; 3, 3' = femur condyles), providing good views of the subpatellar region and the synovial cavity (see arrows). Images acquired in a 9.4-T Varian scanner (Varian, Inc., Oxford, UK) with 100 G/cm gradient coils and a Rapid bird-cage RF coil.

One of the most critical aspects of preclinical MRI is the radio frequency coil which drives both the signal excitation and reception. Because the coil's sensitivity increases as its volume decreases, animal coils are smaller than humans, improving the signal gain significantly while still scanning the anatomical area of interest. Takahashi and collaborators [39] successfully applied MRI technology using a 3D spin-echo pulse sequence in conjunction with spectroscopy to investigate the longitudinal changes in trabecular bone architecture in response to pharmacological interventions in rabbits. Another promising approach is based on the use of ultra-short echo-time (UTE) radial acquisition sequences with hard pulse excitation, which can detect subtle changes in mineral phosphorus and water contents of cortical bone (^31^P and ^1^H), as applied for investigating changes in bone mineralization in *ex vivo *bones from oestrogen deficiency-induced ovariectomised rats [40]. The quantification of water content through UTE-MRI provides a good technique for measuring bone's hydration state *in situ *with great potential for investigating bone metabolic disorders *in vivo*. Figure 4 displays the magnetic resonance (MR) images of rat and mice knees obtained *ex vivo *in a 9.4-T MRI scanner using a 3D gradient echo and fast spin-echo multi-slice acquisitions, respectively.

MRI is extensively used in clinics for assessment of articular cartilage in joint disorders. As in clinical MRI, the MR sequence that best delineated the cartilage from the surrounding tissues, i.e. 3D fat-suppressed spoiled gradient echo, has also been successfully applied for depicting changes in the rat knee of OA models [41]. Moreover, some studies in *ex vivo *rat knees have measured the spatial distribution of T2 relaxation times as a function of the water content and the collagen ultrastructure correlating with the structural integrity of the articular cartilage [42]. These measurements may permit early detection of changes of cartilage matrix integrity, which could lead to osteoarthritis, and thus would be a relevant preclinical model for the development of clinical treatment before any cartilage morphological alterations occur.

Another commonly used approach is the use of contrast agents such as gadolinium (Gd-DTPA, gadopentetate dimeglumine or Magnevist™ Schering, Berlin, Germany). The Gd-DTPA penetrates into cartilage and distributes preferentially to areas of the cartilage in which the GAGs are low. Subsequently, this will induce a decrease in T1 relaxation that reflects the Gd-DTPA concentration and thus the distribution of GAGs. This clinically validated technique is referred to as delayed gadolinium-enhanced MRI of cartilage and has also been investigated in preclinical models [43]. Another application of contrast-enhanced MRI is the assessment of blood flow perfusion and permeability which has important physiopathological relevance as reduced bone blood perfusion is also an indicator of disease progression and severity. Contrast agents like gadolinium (Gd) have been applied for imaging bone blood flow using dynamic contrast-enhanced MRI techniques in animal models of OA [41]. These techniques may be very valuable to assess the healing process and estimate the risk of avascular necrosis.

Finally, ongoing developments in preclinical MRI technology, with the implementation of phased-array coils which by contrast with the traditional single-channel surface coil can increase the signal-to-noise ratio, thereby providing superior image sensitivity covering a specific field of view, have been successfully used for imaging the knee joint in rats [44].

### Nuclear imaging technologies

#### PET/SPECT systems technology

Bone scintigraphy is extensively used as one of the most common diagnostic techniques to investigate bone lesions and metastases in various musculoskeletal conditions and in diagnostic orthopaedic medicine. Further innovations such as single-photon emission computed tomography (SPECT) and positron emission tomography (PET) have allowed the acquisitions of whole-body images of the entire skeleton. They provide increased sensitivity for lesion detection and, importantly, a 3D localization of the radiation emitted by radionuclide imaging agents or biomarkers with very high detection sensitivity down to nano- or picomolar concentration.

The growth in clinical nuclear imaging applications has led to the development of SPECT and PET scanners dedicated for small animal imaging [45,46]. These systems have to cope with the small size of rodents; hence, they have to achieve enhanced spatial resolution and high sensitivity for the targeted biomarker. The application of this technology in preclinical models has a significant scope for non-invasively studying dynamic biological processes at the molecular and cellular level. It provides good functional information through the detection of onset and progression of a given biological process, and can assist in the development of biomarkers and in measuring the effectiveness of new treatments.

SPECT systems record gamma rays directly after radionuclide emission. The system uses a gamma camera to acquire projections data that are detected using a parallel hole collimator. However, most of the preclinical SPECT scanners are equipped with multipinhole collimators to acquire high spatial resolution of foci of gamma-emitting tracers within the subject volume. Then, a tomographic reconstruction of the data is acquired yielding a 3D dataset that can then be manipulated to show any particular axis of the body. PET systems also detect gamma rays that are emitted by a biomarker tracer labelled to a positron emitter (F-18, C-11, N-13, O-15). These radio-nuclides emit positrons which cause two gamma photons to be emitted in opposite directions by annihilation with an electron. The two photons are accepted to be in coincidence if their incidence on a detector pair is within a predefined timing resolution window. The scanner detects these dual emissions 'coincident in time', providing a higher radiation on a given location and thus higher resolution images. This is in contrast to SPECT systems that rely on hardware collimation. The resolution of preclinical PET scanner lies in the range of 1 to 2 mm, but some state-of-the-art dedicated preclinical SPECT systems can provide better resolution capabilities (down to the sub-millimetre range) [43]. SPECT radiopharmaceuticals have a long half-life, and they are routinely produced for clinical nuclear medicine, making SPECT isotopes easily available and more cost-effective than PET tracers. Nevertheless, the high sensitivity of PET tracers and their integration as biomarkers with multi-capability applications makes them very well suited for small animal imaging. Tracers such as ^18^F-FDG, a glucose analogue, and ^18^F-FLT, a pyrimidine analogue are used as biomarkers of tissue metabolic activity and inflammation, and cell proliferation, respectively.

#### PET/SPECT imaging applications

**Figure 5:**
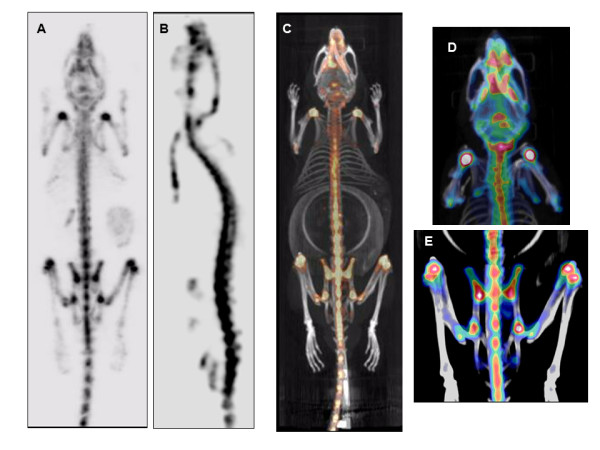
**Combination of micro-SPECT and micro-CT images**. Acquired 3 h post-injection of ^99 m^Tc-MDP (dose 150 MBq i.v.; bone-targeted tracer) demonstrating *in vivo *bone imaging in C57BL/6 mouse. Micro-SPECT data were obtained through helical acquisition over an axial field of view of 100 mm through 60 projections of 30 s. (**A**, **B**) Full body SPECT sagittal and coronal images, respectively, to detect the high MDP uptake within the skeleton with high uptake in joints (knee, shoulders, hip), spine and in the skull. (**C**) Micro-SPECT/CT coronal 3D image of the animal showing the MDP uptake; note the high spatial resolution of the CT acquisition to localise the main areas of Tc-99 m-MDP uptake. (**D**, **E**) Micro-SPECT/CT images of the anterior quarter of the animal, showing the high uptake of the tracer in the skull and shoulders-glenohumeral joint, and the posterior quarter, showing the uptake in the hip joint (femur and sacrum) and the knee joints.

Several studies have successfully reported the use of Tc-99 m-labelled diphosphonate compounds, e.g. methylene diphosphonate (MDP), hydroxymethane diphosphonate and hydroxyethylidene diphosphonate, to detect changes in bone turnover and cartilage composition in osteoarthritis models in rodents [47]. The co-registration of micro-SPECT/micro-CT images allows the detection of high Tc-99 m MDP uptake, depicting areas of high bone turnover, e.g. joints (knees, shoulders), spine and skull (Figure 5). SPECT imaging of articular cartilage in rodents has been reported using *N*-[triethylamonium]-3-propyl-[15]ane-N (NTP) bound to Tc-99 m. NTP has a high affinity for binding to negatively charged sulphate groups of the glycosaminoglycans, a major component of the cartilage matrix, and it has been successfully used for *in vivo *imaging of a cartilage tumoural model in rats [48].

For PET imaging, ^18^F-fluoride (NaF) is being extensively used for assessing bone metabolism, and it has a similar uptake mechanism to ^99 m^Tc-MDP, being absorbed onto bone surfaces. This tracer diffuses well through capillaries reaching bone extracellular fluid where it is rapidly incorporated in the bone hydroxyapatite. Its negligible binding to plasma proteins, rapid blood and renal clearance with the high uptake after injection (40 to 60 min) provides a higher accuracy than SPECT imaging. Similar to MDP, it is taken up preferentially in malignant cancerous bone lesions such as sclerotic metastases and areas of altered osteogenic activity, reflecting an increase in regional blood flow and bone turnover [49] and for detecting bone micro-damage [50] in preclinical models.

^18^F-FDG is another PET tracer that can be used to assess inflammatory activity indirectly in bone. ^18^F-FDG is not specifically targeted to bone, but as a glucose analogue, it provides a sensitive complementary functional biomarker in defining areas of inflammation in the axial and appendicular skeleton. PET scanning with ^18^F-FDG has great potential for assessing fracture healing as it can provide a direct quantitative non-invasive assessment of the metabolic activity in the region of interest and therefore measure bone repair in fracture models [51], providing information on the treatment and prognosis of delayed fracture healing. ^18^F-FDG is also widely used in clinical oncology in musculoskeletal sarcomas, as the tracer is directly taken up into tumour cells and is often used to detect metastases. It has been used successfully to localise and quantify skeletal metabolic activity in the study of preclinical cancer metastasis in bone [52].

### Optical imaging technologies

Optical imaging, including fluorescence and bioluminescence, is becoming an attractive tool for examining and monitoring disease states and to determine therapy effectiveness in living tissues in preclinical models.

#### Fluorescence technology and applications

Fluorescence imaging relies on the detection of light emission of specific fluorophore when excited by appropriate wavelength energy. Several approaches have been described *in vivo*, including the use of non-specific dye tracking (e.g. for detection of non-specific inflammatory sites), the use of fluorophores tagged-antibody-based targets for specific cell molecules and/or metabolic pathways and the development of fluorescence reporter proteins (e.g. GFP) for the detection of gene expression. Targeted fluorescence imaging of arthritic joints has shown an increase in fluorescence signal in the arthritic joints in disease models. This is in part associated with increased blood perfusion and vascular leakiness, which are recognised to occur in areas of inflamed synovium [53]. *In vivo *targeting of specific key components identified during the inflammatory process such as the labelling of the F4/80 antigen on macrophages has been achieved [54]. Similarly, other components of the inflammatory cascade can be traced *in vivo*. For example, E-selectin-targeted *in vivo *imaging is a quantifiable method of detecting endothelial activation. E-selectin or endothelial adhesion molecule-1 is a 115-kDa glycoprotein induced on endothelial cells in response to pro-inflammatory cytokines involved in rheumatoid arthritis such as interleukin-1β and tumour necrosis factor alpha (TNF-α) [55]. E-selectin has been well validated as a potential biomarker of disease activity in rheumatoid arthritis [56].

**Figure 6:**
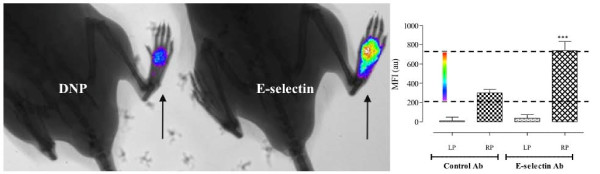
**Specific anti-E-selectin targeted fluorescent signal co-registered with X-ray imaging**. Following injection of either anti-E-selectin or anti-DNP (control) antibodies labelled with Dylight 750 nm NIR fluorophore (Thermo Fisher Scientific Inc., Rockford, IL, USA) at a dose of 5 μg i.v., paw swelling was induced by intraplantar injection of murine TNF-α into the right paw (RP) in C57/BL6 mice (marked by arrows) (*n *= 4 to 6). Mean fluorescence signal (MFI) quantified at the 8 h time point is shown for different groups of mice. The mean background intensity from control and anti-E-selectin-targeted animals was subtracted. In the left-hand panel, the corresponding fluorescent image overlaid onto a co-registered X-ray. The colour wheel to depict signal intensity has been adjusted to the range as shown on the graph.

Anti-E-selectin antibody labelled with NIR fluorophore has demonstrated specific signal increases compared to control antibody in a mouse model of paw swelling induced by the intra-plantar injection of TNF-α. This has also been demonstrated in acute collagen-induced arthritis, a widely used model of RA. Mapping of fluorescent E-selectin-specific signal in difference to the signal returned from fluorescently labelled control antibody is demonstrated in Figure 6[57]. Utilising fluorescent compounds to label antibodies allows for a more targeted and specific approach to identify different components of disease, quantify levels of inflammation and assess the effect of novel therapies. Similarly, activity-based fluorophores where signal becomes amplified at the site of inflammation have also been used successfully in mouse models of arthritis demonstrating increases in signal intensity in the injured joint [58]. This type of approach may be particularly useful for optical imaging in OA, since local perturbations in proteolytic activity may be small and require amplification for adequate signal detection.

Fluorescence is also extensively used to image the skeleton, with a multitude of commercially available bone specific fluorophores (e.g. OsteoSense™ from PerkinElmer, Waltham, MA, USA), fluorescently labelled alendronate from Caliper^®^LifeSciences (Hopkinton, MA, USA) and BoneTag™ from LI-COR^®^Biosciences (Lincoln, NE, USA) [59], which are incorporated in the calcified bone matrix at spots with high bone turnover and are therefore good indicators of bone remodelling in sites of bone damage such as fractures and cancer-induced osteolytic/blastic lesions [60].

#### Bioluminescence technology and applications

Bioluminescence imaging is based on the detection of photons produced after an enzymatic reaction in living organisms. It is commonly used in reporter gene assays, where the promoters of genes under study have been linked to the luciferase gene, which in the presence of its substrate (luciferin) generates light. The most commonly used luciferase enzyme used for bioluminescence imaging purposes comes from the firefly (emitting light around 560 nm). This approach requires the luciferase enzyme to be transfected into the living cells and must be tightly controlled with the appropriate promoter. Also, a substrate needs to be delivered where the enzyme may be expressed. This may be challenging for certain organs and may affect the dynamics of the emission response due to different pharmacokinetic profiles related to altered tissue properties. Despite these conditions, its high specificity, low background signal, high signal-to-noise ratio and the ability to study gene expression over the lifetime of the animal are reasons why this technology is used extensively for *in vivo *imaging studies. Bioluminescence imaging has been successfully utilised to study the development of arthritis in transgenic mouse models [61], and because of its high sensitivity, it is also used widely in bone metastasis studies, to monitor the development and progression of luciferase positive tumour cell lines within the whole body [62].

## Challenges for *in vivo *preclinical imaging

The implementation of non-invasive imaging technologies provides a very useful avenue for more rapid, efficacious and cost-effective use and characterisation of animal disease models. They represent a good alternative assessment tool for the invasive techniques and histological or biochemical assays that are already used extensively. By combining different imaging modalities, accurate quantitative and qualitative structural and functional data targeting molecular mechanisms can be acquired (Table 1).

**Table 1:** Main features of current imaging modalities for in vivo preclinical musculoskeletal research

Imaging modality	Resolution	Sensitivity	Imaging time	Application	Radiation	Detection depth of view	Limitations
Micro-CT	10 μm	m-cmol	Seconds to minutes	Excellent contrast for mineralized tissue	X-rays	No limit	Radiation exposure
				Mostly anatomical imaging			Long acquisition times
MRI	50 to 100 μm	μ-mmol	Minutes to hours	Excellent contrast tissue resolution -anatomical and functional Imaging	No	No limit	High investment infrastructure and running cost
							Long acquisition time
							Expert operator
PET	1 to 2 mm	p-nmol	Minutes	Functional imaging	Gamma radiation	No limit	Short half-life PET tracer (requirement cyclotron unit)
							High cost of tracers
							High investment infrastructure
SPECT	<1 mm	p-nmol	Minutes	Tomographic functional imaging	Gamma radiation	No limit	Limited sensitivity
							High investment infrastructure
Fluorescent (optical imaging)	1 to 2 mm	p-nmol	Seconds to minutes	Functional imaging	Fluorescent emission	<1 to 10 cm	Not translated into clinical modality
Bioluminescence (optical imaging)	1 to 2 mm	p-nmol	Seconds to minutes	Functional imaging	Light emission	<10 cm	Not translated into clinical modality
							Injection of substrate

The ability to image live animals is one of the most important advantages of these technologies. But this also represents a major challenge as biological motion, not only gross body movement but also that induced by breathing and cardiac activity, affects the resolution of the images. Animals are imaged under anaesthesia, which helps to restrain their gross motion, but there is still the need to control cardiac and respiratory motion. Moreover, anaesthesia has undesirable effects interfering with the body temperature control and also respiratory rates. Close monitoring is required for refining the efficacy of preclinical testing and ensuring the standardisation and repeatability of studies.

Another important challenge is the small size of animal specimens that require high resolution and sensitivity in smaller fields of view and also take into account the physiological motions of the animals. The implementation of gating methods has markedly improved the acquisitions, minimising any interference effects due to the physiological movement. Gating (prospective or retrospective, if image acquisition is acquired simultaneously or processed post-acquisition) is already implemented in most of the preclinical *in vivo *imaging systems using specialised software. The benefits of applying gating during image acquisition have been well reported in micro-CT imaging of rodents [13]. While such motion artefacts may not be so relevant when imaging appendicular skeleton, their effects are significant when imaging the skull and axial skeleton.

Post-image processing is also of paramount importance, especially when integrating data acquired through different modalities and to correct for the posture and shape variability between different animals or within the same animal through a follow-up study. While analysing techniques used for these multidimensional image datasets are quite complex and are not easily interchangeable, many software tools have been developed (e.g. Amira, ImageJ, OsiriX; see review [63]) providing facilities for image registration, 3D surface-rendering and image segmentation. To integrate different datasets, the most commonly used technique is atlas registration, whereby individual acquisitions are registered to an idealised expert-defined atlas based on prior images. Recently, Baiker and collaborators [64] developed a fully automated method for atlas-based whole-body segmentation from low-contrast micro-CT data which effectively allowed intra- and inter-subject registrations using data acquired *in vivo*, with remarkable accuracy, overcoming large variations in posture and shape. Snoeks and collaborators [65] nicely demonstrated the strength of multi-modality imaging by combining the use of bioluminescence and micro-CT to image bone metastases *in vivo*.

With the increasing demand to acquire higher temporal and spatial resolution images, animal welfare is an important consideration, especially the cumulative effect of any radiation dose on the animal's well-being and physiological status. This is of most concern in longitudinal studies where the cumulative radiation dose can be extremely high. The effect of ionizing radiation in rodents has been well studied after micro-CT. The lethal dose in mice, which is typically expressed as LD_50/30 _(whole-body radiation dose that would kill 50% of exposed animals within 30 days) is in the range 5 to 7.6 Gy. The typical X-ray whole-body radiation dose for a 3D micro-CT scan ranges from 0.017 to 0.78 Gy [4]. These sub-lethal doses of radiation are unlikely to really compromise the animal using *in vivo *micro-CT for longitudinal studies. In the case of musculoskeletal applications, higher resolutions may be required, but it is likely that only a small area of the animal will be imaged. The exposure to radiation may be an issue to be considered in oncological *in vivo *studies as the dose is perhaps more likely to have an effect on the growth of the tumours to be imaged [66] and also could influence other biological pathways affecting bone remodelling [67].

The potential of small animal imaging goes far beyond anatomical studies, and by combining all the different imaging modalities, functional and anatomical analyses can be integrated. While PET and SPECT can provide excellent sensitivity for functional procedures through labelled biomarkers, combination with CT imaging allows for accurate spatial correlation of the biomarker within the body. Similarly, other multi-modality approaches such as PET/MRI are under development to increase the spatial and temporal resolution of the PET and the sensitivity of MRI [68].

With the increasing availability of new tracers as biomarkers to target specific musculoskeletal disorders, data from PET/SPECT or optical imaging combined with CT will play a key role in translational bone and cartilage research. Similarly, optimization of the micro-CT system is likely to lead to more sensitive detectors and will provide higher spatial resolution with shorter acquisition times, reduced radiation dose with improvements in gating acquisitions and interactive reconstruction. These will undoubtedly reinforce the use of these techniques for preclinical research into bone and cartilage disorders.

## Conclusion

In conclusion, the increasing use of imaging technologies in animal models supports its prominent role in translational research for bone and joint disorders. With ongoing developments in micro-CT and MRI preclinical scanners, imaging of bone and cartilage structures can be achieved down to the micro-structural level, effectively visualising trabecular bone structure and cartilage composition. On the other hand, the implementation of PET and SPECT and optical imaging in the musculoskeletal preclinical field provides significant potential for investigating the dynamics of bone and joint biological processes at the molecular and cellular levels with the ability to monitor the effectiveness of novel therapeutic targets. New PET and fluorescent tracers are being developed as biomarkers for specific disorders that can be utilised for testing the efficacy of new drugs and validating their safety at early stages of drug development. Ongoing research is focussing on integrating different imaging modalities with the aim of facilitating successful translational applications from anatomical to functional endpoints, to improve the efficacy in musculoskeletal preclinical studies.

## Competing interests

The authors declare that they have no competing interests.

## Authors' contributions

JLT performed some of the reported animal experiments, reviewed current literature, provided the concept for the review and wrote the manuscript. MK helped in the imaging experiments, provided valuable intellectual advice on the imaging technology and critically reviewed the manuscript. LLG provided valuable input in the fluorescence technology and critically edited the manuscript. TV provided some of the tissue samples for the study, gave valuable intellectual input particularly on joint disorders and critically reviewed the manuscript. MWA and WG critically reviewed the manuscript and supported the conception of this review. All the authors read and approved the final manuscript.
